# Re-establishing immune tolerance in multiple sclerosis: focusing on novel mechanisms of mesenchymal stem cell regulation of Th17/Treg balance

**DOI:** 10.1186/s12967-024-05450-x

**Published:** 2024-07-15

**Authors:** Huiru Hu, Hui Li, Ruoyu Li, Peidong Liu, Hongbo Liu

**Affiliations:** 1 Department of Neurology, The First Affiliated Hospital of Zhengzhou University, Zhengzhou University, Zhengzhou, 450000 Henan China; 2 Department of Neurosurgery, First Affiliated Hospital of Zhengzhou University, Zhengzhou University, Zhengzhou, 450000 Henan China; 3 Translational Medicine Center, First Affiliated Hospital of Zhengzhou University, Zhengzhou, 450000 Henan China

**Keywords:** Mesenchymal stem cell, T-helper 17 (Th17) cell, Regulatory T cell (Treg), Multiple sclerosis, Immune tolerance

## Abstract

The T-helper 17 (Th17) cell and regulatory T cell (Treg) axis plays a crucial role in the development of multiple sclerosis (MS), which is regarded as an immune imbalance between pro-inflammatory cytokines and the maintenance of immune tolerance. Mesenchymal stem cell (MSC)-mediated therapies have received increasing attention in MS research. In MS and its animal model experimental autoimmune encephalomyelitis, MSC injection was shown to alter the differentiation of CD4^+^T cells. This alteration occurred by inducing anergy and reduction in the number of Th17 cells, stimulating the polarization of antigen-specific Treg to reverse the imbalance of the Th17/Treg axis, reducing the inflammatory cascade response and demyelination, and restoring an overall state of immune tolerance. In this review, we summarize the mechanisms by which MSCs regulate the balance between Th17 cells and Tregs, including extracellular vesicles, mitochondrial transfer, metabolic reprogramming, and autophagy. We aimed to identify new targets for MS treatment using cellular therapy by analyzing MSC-mediated Th17-to-Treg polarization.

**Graphical Abstract:**

**Figure:**
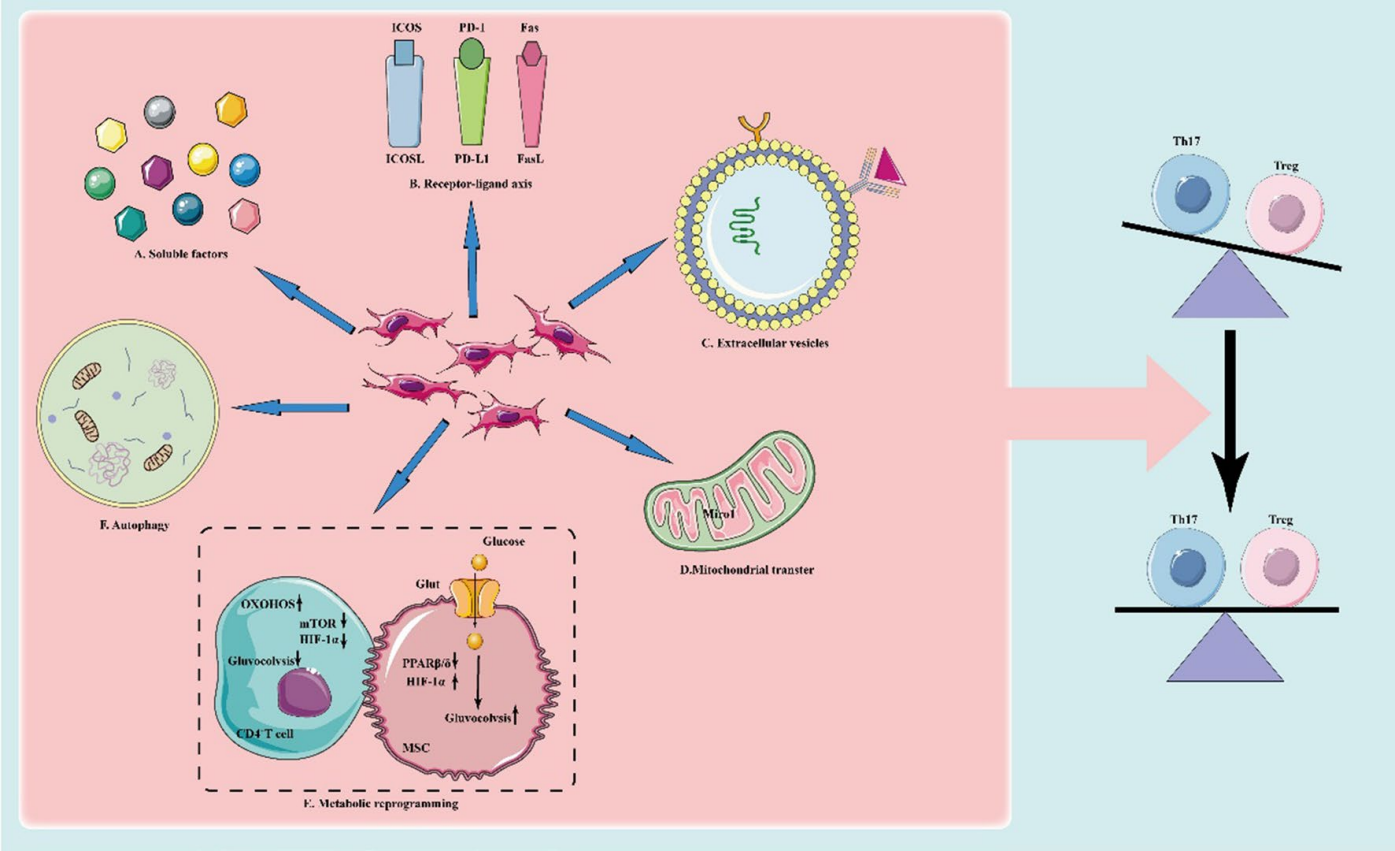
Pathways of mesenchymal stem cells for regulating Th17/Treg homeostasis

## Introduction

Multiple sclerosis (MS) is an inflammatory immune-mediated disease characterized by aberrant, pro-inflammatory CD4^+^T cells in the central nervous system (CNS) that cause non-traumatic disability in young adults [1, 2]. MS is traditionally divided into three main clinical types: relapsing–remitting MS (RRMS), primary progressive MS (PPMS), and secondary progressive MS (SPMS) [3, 4]. Previous studies have shown that MS is characterized by immune dysregulation, mainly driven by myelin-specific autoreactive CD4^+^T cells, and is closely related to immune dysfunction, transitional activation of immune cells, and an imbalance in the ratio of immune cell subpopulations [5–7]. An imbalance between T-helper 17 (Th17) cells and regulatory T cells (Tregs) plays a key role in the pathogenesis of MS [8–10]. When peripheral immune tolerance is disordered, autoreactive CD4^+^T cells in the lymph nodes, including T-helper 1 (Th1) cells and Th17 cells, are activated and become aggressive effector cells, including T-helper 1 (Th1) cells and Th17 cells [1, 11]. The Th17 cells disrupt the blood–brain barrier (BBB) by secreting interleukin (IL)-17A [12], inducing the expression of inflammatory cytokines and chemokines and recruiting other immune cells (lymphocytes, macrophages, and neutrophils) to the CNS [2, 13, 14]. In the CNS, autoreactive CD4^+^T cells are reactivated and amplified by IL-23 and IL-1β (produced by resident microglia and infiltrating inflammatory monocytes) and can be polarized to produce excess Th17 cells [11]. Th17 cells overactivate microglia in a positive feedback loop and assist B cells in antibody production [15]. Subsequently, these immune cells release different pathogenic cytokines that cause an inflammatory cascade and damage oligodendrocytes, ultimately leading to axonal degeneration and neuronal dysfunction [16, 17]. In contrast, Tregs have immunosuppressive functions and inhibit effector cell-mediated inflammatory immune responses to maintain peripheral immune tolerance through secretion of anti-inflammatory factors, such as IL-10, transforming growth factor-β (TGF-β), and IL-35 [1]. Additionally, Tregs can inhibit the inflammatory immune response mediated by activated dendritic cells and pathogenic B cells [1, 11]. Therefore, peripheral immune tolerance is disrupted when Tregs are defective and/or when effector cells are resistant to Tregs [1, 18]. In patients with MS, Treg cell defects are mainly observed as changes in cell quantity, subset changes, migration, and dysfunction, and Tregs are unable to suppress the inflammatory response triggered by Th17 cells, ultimately causing an autoimmune response [18, 19]. Thus, in patients with MS, the skewed ratio of Th17/Treg cells seems to be the main driver of immunopathology, leading to disruption of the immune response and immune tolerance balance in vivo [20, 21]. Currently, there are many immunotherapies to restore the balance of Th17/Treg in MS, such as various disease-modifying therapies (DMT), immunosuppressive drugs, including interferon beta (IFN-β) [22], glatiramer acetate (GA) [23], teriflunomide, and fingolimod, and various monoclonal antibodies based on cell depletion therapy [22, 24–29]. These therapies reduce the recurrence rates and lesion activity by targeting and blocking immune activation and inflammation [2, 25, 27]. However, they also suppress the systemic immune response and the effect of these drugs on counteracting the inflammatory cascade in patients with MS [30, 31].

Experimental autoimmune encephalomyelitis (EAE) is an antigen-driven autoimmune model in which immunization against myelin autoantigens elicits strong T cell responses that initiate its pathology with CNS myelin destruction [32]. Similarly, an inappropriate immune response of Th17 cells and dysfunction of Treg cells are responsible for dysregulated EAE immunity, inflammatory response, oxidative stress, and attack on myelin self-basic protein (MBP) [14, 33]. Therefore, upregulation of anti-inflammatory Treg cells, inhibition of pro-inflammatory Th17 cells, and restoration of the balance of T-cell responses are ideal strategies for EAE treatment. For example, ginsenoside Rd, Rapamycin, and others alleviate the inflammatory response in EAE by altering the Th17/Treg balance [34–36].

Mesenchymal stem cells (MSCs) are multipotent stromal cells that exist in many human tissues and are characterized by their rapid expansion in vitro [37, 38]. MSCs originate from a variety of organs and tissues, such as bone marrow (BM), adipose tissue, muscle, umbilical cord (UC), and placental tissue [39, 40]. MSCs are considered a powerful tool for controlling MS progression and restoring immune tolerance owing to their powerful immunomodulatory effects and lower immunogenicity [41, 42]. Currently, MSCs are used clinically for the prevention and treatment of MS and other autoimmune diseases (such as rheumatoid arthritis and systemic lupus erythematosus) [37, 38, 40, 43]. Numerous pre-clinical studies have demonstrated that MSCs can regulate the differentiation of CD4^+^T cell subsets by limiting Th17 cell proliferation and promoting Treg production and immunosuppressive capacity, thereby regulating immune disorders, counteracting autoimmune responses in EAE, and ultimately maintaining immune tolerance [44]. Furthermore, allogeneic MSC transplantation is safe, feasible, and potentially effective in clinical trials for the treatment of immune-related diseases [41]. Thus, a deeper understanding of the potential mechanisms of MSC-mediated Th17/Treg homeostasis is necessary to help develop novel MSC-based therapies for more targeted immune-molecular therapies and improve the possibility of utilizing MSCs as cell therapy in the clinical treatment of MS.

In this review, we discuss the skewed ratio between Th17 cells and Tregs in MS/EAE and the effect of MSCs in regulating Th17/Treg balance. The main pathways/molecular mechanisms of MSCs in regulating the Th17 cell and Treg balance, such as extracellular vesicles (EVs), mitochondrial transfer, metabolic reprogramming, and autophagy, will reveal new targets of MSCs for MS.

## The imbalance of Th17 and Treg in multiple sclerosis

The disruption of immunologic tolerance and the active infiltration of myelin antigen-sensitive immune cells into the brain parenchyma through the BBB are essential pathogenic mechanisms in MS [13, 45]. Importantly, the increased pro-inflammatory effects of Th17 cells and the diminished immunosuppressive capacity of Tregs are crucial factors driving the loss of immune tolerance in MS [14]. Th17 cells trigger the inflammatory cascade by secreting large amounts of pro-inflammatory cytokines and chemokines. Tregs inhibit the immune response and maintain self-tolerance by promoting the secretion of immune suppressive cytokines, ultimately protecting against worsening MS disability [18].

### Th17 cells augmented pro-inflammatory effects

Excessive proliferation and activation of Th17 cells is an important mechanism leading to the development of MS [2, 13]. Numerous studies have shown that the quantity of Th17 cells and IL-17 is elevated in the blood and cerebrospinal fluid (CSF) of patients with MS and is positively associated with disease activity and relapse frequency [46, 47]. Th17 cells mediate neuroinflammation in MS by releasing various pro-inflammatory cytokines and chemokines [13, 48]. For example, IL-17, a central mediator of the pro-inflammatory effects of Th17 cells, enhances the activation of matrix metalloproteinase-3 (MMP-3) and attracts neutrophils to the site of inflammation, disrupting the BBB and leading to infiltration of Th17 cells and other immune cells into the CNS [26, 49]. In addition, C–C chemokine receptor 6 (CCR6) is a key mediator that drives Th17 cells to participate in the immune response and is critical for Th17 cell migration to the site of inflammation [50]. In the CNS of EAE mouse models, endothelial barriers are rich in CCL20, a CCR6 ligand [47, 51]. CCL20 is constitutively expressed in epithelial cells of the choroid plexus. It attracts CCR6, and this interaction allows Th17 cells to cross the epithelial barrier of the choroid plexus and enter the CSF through CCR6-mediated signals in EAE mice [47, 51]. Thus, the initial trigger of inflammation in EAE mice is CCR6-dependent autoreactive Th17 cell infiltration into the uninflamed CNS. Unlike other Th17 cytokines, granulocyte–macrophage colony-stimulating factor plays an important role in mediating myeloid cell infiltration during persistent neuroinflammation by impairing the accumulation of tissue-invading phagocytes [52–55], which are the primary drivers of immunopathology in MS [42–45]. Interestingly, a novel subpopulation of Th17 cells, defined as Th1-like Th17 cells (Th17.1), has recently been identified. Th17.1 cells co-express the transcription factors RORC and T-bet (a major regulator of Th1 differentiation) and share the inflammatory and pathogenic characteristics of Th1 and Th17 cells [56]. This combination further disintegrates the BBB and relieves lymphocyte migration [17]. In addition, high expression of very late antigen 4 (VLA-4) on the surface of Th17.1 cells promotes CNS infiltration [17]. Previous results have shown that Th17.1 cells were significantly increased in patients with acute relapsing MS and involved in MS pathogenesis through dual expression of IFN-γ and IL-17A [26]. Several studies have shown that Th17.1 can cross the BBB and enhance neuroinflammation by stimulating the secretion of IL-17 and CCR6 in EAE [13, 17]. In addition, Th17 cells can secrete other cytokines, such as IL-6, IL-21, IFN-γ, IL-22, and IL-23, that enhance the immune response in patients with MS [2, 47].

### Tregs-weak protective effects

Tregs are a classical type of inhibitory T cell that negatively regulates immune cell function. They primarily suppress the pro-inflammatory response of effector T cells and maintain immune tolerance in the periphery via multiple soluble mediators (including IL-10, IL-35, and TGF-β) and cell surface molecules (including IL-2 receptor alpha chain/IL-2RA [CD25] and cytotoxic T-lymphocyte-associated antigen 4) [57]. Previous studies have demonstrated that Treg defects in patients are mainly observed as changes in cell quantity, subset changes, migration, and dysfunction [58, 59]. For example, a previous study reported that the percentage of Tregs in the peripheral blood of patients with MS is significantly reduced and is associated with clinical disease severity [60]. In addition, a previous study indicated that the number of Tregs in the CSF, but not in peripheral blood, is elevated in patients with MS [61]. In contrast, alterations in Treg cell subset proportions and Treg dysfunction are more pronounced in patients with MS [62]. For example, the effector function of CD4^+^CD25^hi^ Tregs in peripheral blood is notably downregulated in patients with MS [63]. Moreover, CD46-mediated type 1 Treg (Tr1) is another major Treg defect, and compared with healthy controls, there were striking defects in IL-10 secretion among Tr1 cells with CD46 co-stimulation in MS [64–66]. An in vitro experiment showed that CD46 is a newly defined co-stimulatory molecule that can induce the Tr1 phenotype with considerable amounts of IL-10 secretion [67, 68]. A recent in *vitro* study suggested that defects in Treg suppressor molecules, such as reduced IL-10 production and genetic variations in CD25, are related to MS [69, 70]. Additionally, Fritzsching et al. reported that Tregs do notaccurately infiltrate the CNS during the progression of MS, while brain biopsies from patients with MS showed a lack of FoxP3 expression in 30% of lesions [71]. In addition, Fas, a cellular apoptotic pathway receptor, is upregulated on Tregs in MS brain biopsies, suggesting increased susceptibility to apoptosis [71]. These findings suggest that Tregs are restricted from migrating into the neuroinflammatory niche and undergoing apoptosis during the early stages of infiltration [18, 71].

Currently, there are numerous immunotherapies available to restore the Th17/Treg balance in MS [2]. For example, an in vitro study suggested that dimethyl fumarate (DMF) was shown to significantly reduce the relative and absolute number of Th17 cells [72], and anti-CD20 monoclonal antibodies hindered Th17 cell differentiation through direct (depletion) and indirect (reduced B cell activation) mechanisms, thereby inhibiting the pro-inflammatory effects of Th17 cells in MS. However, enhancing the ability of Tregs to maintain self-tolerance appears to be an alternative therapy for MS clinically and includes IFN-β, glatiramer acetate (GA; Copaxone), fingolimod (Gilenya), and teriflunomide (Aubagio) [71, 73]. These therapies have been clinically shown to alleviate the clinical symptoms of MS by increasing the number of Tregs and their immunosuppressive function [73–75]. These DMTs and various monoclonal antibodies based on cell depletion therapy have alleviated the Th17/Treg imbalance in patients with MS to some extent [76]. However, these drug therapies are nonspecific and suppress the systemic immune system with an increased risk of infection, tumors, and other adverse effects [76, 77].

## Mesenchymal stem cells regulate the potential mechanisms of Th17/Treg homeostasis

Based on published and ongoing clinical trials and laboratory research, MSCs have demonstrated an ability to modulate the differentiation of CD4^+^T cell subsets, such as through inhibition of Th17 cell proliferation, induction of Treg production, and immunosuppressive functions [78, 79]. Therefore, re-establishing the balance of Th17/Treg cells and regulating immune disorders in EAE will ultimately restore immune tolerance and maintain immune homeostasis [78]. For example, bone marrow-derived MSCs (BM-MSCs) inhibit the differentiation of naïve T cells into Th17 cells and suppress the secretion of IL-17 and IL-22 [80, 81]. Similarly, infused BM-MSCs inhibit the progression of EAE in vivo by reducing the secretion of IL-17 and IL-23 [79]. Interestingly, owing to the strong plasticity of Th17 cells, they possess the ability to transdifferentiate into Foxp3IL-10 Tr1 and suppress immune responses in EAE [82, 83]. Furthermore, BM-MSCs were found to promote FoxP3 expression with increased IL-10 secretion and suppress RAR-related orphan receptor (ROR) C expression with reduced IL-17 and IL-22 in differentiated Th17 cells [80]. In contrast, MSCs enhance the immunosuppressive ability of Tregs. For instance, MSCs induce FoxP3 expression by secreting indoleamine 2, 3-dioxygenase (IDO), which increases the proportion of Tregs in the spleen of EAE patients, leading to a reduction in the clinical score and severity of EAE [84]. Meanwhile, in vitro experiments have shown that co-culture of T cells and MSCs can significantly upregulate FoxP3 expression in Tregs and increase the proportion of Tregs [85].

**Fig. 1:**
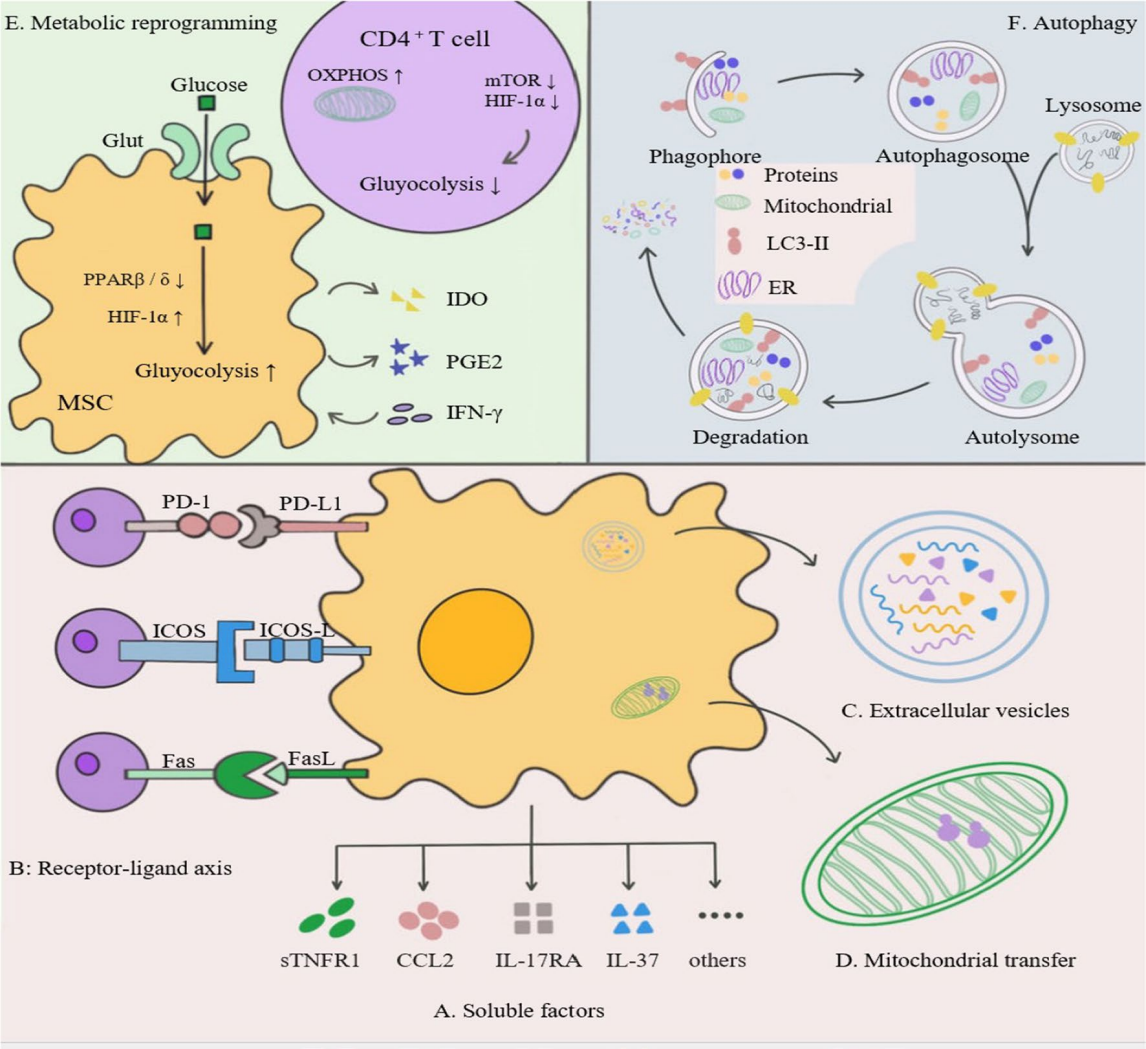
Schematic diagram of MSC-mediated reconstruction of the normal Th17/Treg balance. From the bottom-up: **A** Soluble factors: sTNFR1, CCL2, IL-17RA and IL-37; **B** Receptor-ligand axis: PD-L1/PD-1, ICOSL-ICOS, FAS-FASL;** C** Extracellular vesicles: miRNAs, proteins, tolerance molecules, etc.; **D** Mitochondrial translocation: inhibiting the glycolytic process in CD4^+^ T cells and Th17 cells, and enhancing the oxidative phosphorylation process that induces Treg generation; **E** Metabolic reprogramming: by enhancing the glycolytic metabolism of MSCs as well as inhibiting the glycolytic metabolic process of CD4^+^ T cells; and **F** Autophagy: The autophagic process of MSCs mediates the differentiation of MSCs to CD4^+^ T cells and their subpopulations. Through the above pathways, MSCs inhibit Th17 cell production and their pro-inflammatory effects, induce Treg proliferation and immunosuppressive functions, and thus regulate the Th17/Treg balance

Accordingly, the therapeutic strategy to restore the Th17/Treg balance in MSCs is a novel immunomodulatory strategy aimed at re-establishing immune tolerance. In view of the extensive in vivo and in vitro studies on MSCs, we attempted to elucidate the potential mechanisms of MSC-mediated regulation of Th17/Treg homeostasis from six major pathways (Fig. 1), including soluble factors, intercellular contacts, and EVs in the hope of contributing to the expansion of MSC therapy into an increasing number of immune-molecular therapies [42].

### Soluble factors

MSCs can reverse the Th17/Treg skew through a paracrine pathway. In vitro and *vivo* findings have shown that this effect is mainly mediated by a variety of soluble factors secreted by MSCs, including cytokines, growth factors, chemokines, and other immunomodulatory factors [86–88]. An in vivo study suggested that MSCs derived from skin tissue could produce large amounts of soluble TNF receptor 1 (sTNFR1), which blocks TNF-α-mediated signaling and function by binding TNF-α, inhibiting RORγt expression and Th17 cell production, and ultimately, significantly improving clinical scores in EAE [89]. TNF-α has also been shown to drive IL-17 production and differentiate T cells into the Th17 phenotype [90]. Moreover, Moutih et al. found that MSC-derived CCL2 binds to CCR2 expressed by Th17 cells, which inhibits STAT3 phosphorylation and reduces Th17 cell production in EAE mice, ultimately attenuating the severity of EAE. MSC-driven MMP hydrolytic processing of the CCL2 protein subsequently converts CCL2 from an agonist to an antagonist of T cell chemotaxis and activation, thereby inhibiting the enhanced inflammatory effects of Th17 cells in EAE [91]. Additionally, IL-17RA expressed by MSCs enhances the expression of other immunosuppressive mediators (such as VCAM1, intercellular adhesion molecule [ICAM]-1, and programmed death ligand 1 [PD-L1]) and inhibits the proliferation and differentiation of Th17 cells. Sivanathan et al. injected IL-17RA-/- MSCs into EAE mice and found that IL-17RA-/- MSCs were unable to reduce the number of Th17 cells in the lymph nodes of mice and attenuated the inflammatory response in vivo. In addition, the study reported that MSCs induce Treg production in an IL-17RA-dependent manner [92]. Recent studies have shown that MSCs secrete IL-37, a dual-function cytokine, in both intracellular and extracellular forms, which mediates Th17 /Treg homeostasis [93]. Intracellularly, MSC-secreted IL-37 is cleaved by caspase-1 and binds to phosphorylated Smad-3 to form an IL-37-Smad3 complex, which can block transcription of pro-inflammatory cytokines and chemokines such as IL-17, IL-1α, IL-6, TNF, and CXCL2, ultimately reducing the pro-inflammatory effect of Th17 cells and attenuating the severity of EAE mice [94]. Transgenic expression of IL-37 reduces inflammation and prevents neurological defects and myelin loss in EAE mice by acting via IL1-R5/IL1-R8 [95]. Therefore, IL-37 is a promising novel target for future MS therapies. Other soluble factors such as IDO [84, 96], TGF-β [97], prostaglandin E2 (PGE2) [98], hepatocyte growth factor [99], human leukocyte antigen (HLA)-G5 [100], heme oxygenase-1 [101], and inducible nitric oxide synthase may also be involved in the regulation of Th17/Treg homeostasis. Table 1 summarizes the major soluble factors that regulate Th17/Treg homeostasis in MSCs.

**Table 1:** The major soluble factors that regulate Th17/Treg homeostasis in MSCs

The type of soluble factors	Sources of MSCs	In vitro or in vivo	Effects of MSCs on Th17/Treg	Authors	References
sTNFR1	Skin	In vivo	Inhibit RORγt expression and Th17 cell production, and ultimately, significantly improving clinical scores in EAE	Ke et al.	[84]
CCL2	BM	In vivo	Bind to CCR2 on the surface of Th17 cells, inhibits STAT3 phosphorylation in Th17 cells, and reduce the production of Th17 cells in EAE mice	Rafei et al.	[86]
IL-17RA	AD	In vivo	Inhibit the proliferation and differentiation of Th17 cells and enhance the expression levels of other immunosuppressive mediators such as: VCAM1, ICAM1 and PD-L1	Kurte et al.	[87]
IL-37	H-hPDLSCs-CM	In vivo	Binds to phosphorylated Smad-3 to form IL-37-Smad3 complex, reducing secretion of pro-inflammatory factors such as: IL-17, IL-1α, IL-6, TNF and CXCL2	Giacoppo et al.	[89]
IDO	Murine endometrial-derived MSCs	In vivo	Reduced Th1 and Th17 cells both in the periphery and CNS, whereas IL-10-secreting T CD4 + lymphocytes were increased, ultimatly suppressing EAE scores	Polonio et al.	[92, 93]
PGE2	BM	In vitro	Inhibit IL-17A secretion and Th17 cell production via an EP4-mediated, contact-dependent mechanism	Duffy et al.	[95]
TGF-β	Unknown	In vitro	Inhibit Th17 cell production mediated by dendritic cells, induce the differentiation of conventional CD4 CD25^++−^ T cells into Foxp3 Treg cells	Favaro et al.	[94]

*AD* adipose tissue, *BM* bone marrow, *H-hPDLSCs-CM* human periodontal ligament stem cells conditioned medium, *MSCs* mesenchymal stem cells, *EAE* experimental autoimmune encephalomyelitis, *sTNFR1* soluble TNF receptor 1, *IDO* indoleamine 2, 3-dioxygenase, *PGE2* prostaglandin E2, *EP4* PGE2 receptor 4, *VCAM* vascular cell adhesion protein, *ICAM1* intercellular adhesion molecule, *PD-L1* programed death ligand 1

### Receptor-ligand axis interactions

MSCs regulate downstream pathways in CD4^+^T cells by interacting with CD4^+^T cell surface receptors and/or ligands, which can affect CD4^+^T cell activation, differentiation, and induction of Treg production [91–93]. Kim et al. demonstrated that human palatine tonsil-derived MSCs (T-MSCs) directly inhibit STAT3 phosphorylation in CD4^+^T cells via the PD-L1/PD-1 axis, leading to a reduction in Th17 cell production in vivo [102]. Additionally, the Fas-FasL-mediated apoptotic signaling pathway is involved in the immunomodulation of MSCs. Yang et al. reported that gingival-derived MSCs (GMSCs) couple to T cells via the Fas/FasL pathway, which simultaneously induced T cell apoptosis, inhibited Th17 cell differentiation, and induced Treg cell production, which ultimately attenuated inflammation in vitro [103, 104]. A possible mechanism is that Fas induces T cell recruitment by BM-MSCs by regulating the secretion of monocyte chemotactic protein 1, which in turn leads to apoptosis of effector T cells. The subsequent fragmentation of apoptotic T cells can trigger the production of high levels of TGF-β by macrophages, leading to the upregulation of Tregs and thus inducing immune tolerance in vivo [105]. In addition, Lee et al. demonstrated that BM-MSCs co-cultured with CD4^+^T cells via Transwell induced the differentiation of Tregs and showed a correlation with the ICOS/ICOSL axis. This induction of Treg differentiation is mainly due to the activation of the PI3K-AKT signaling pathway in CD4^+^T cells, followed by AKT-mediated activation of glycogen synthase kinase-3 through Toll-like receptor ligation, promoting IL-10 production, FoxP3 expression, and ultimately the induction of Treg differentiation [106].

### Extracellular vesicles

**Fig. 2:**
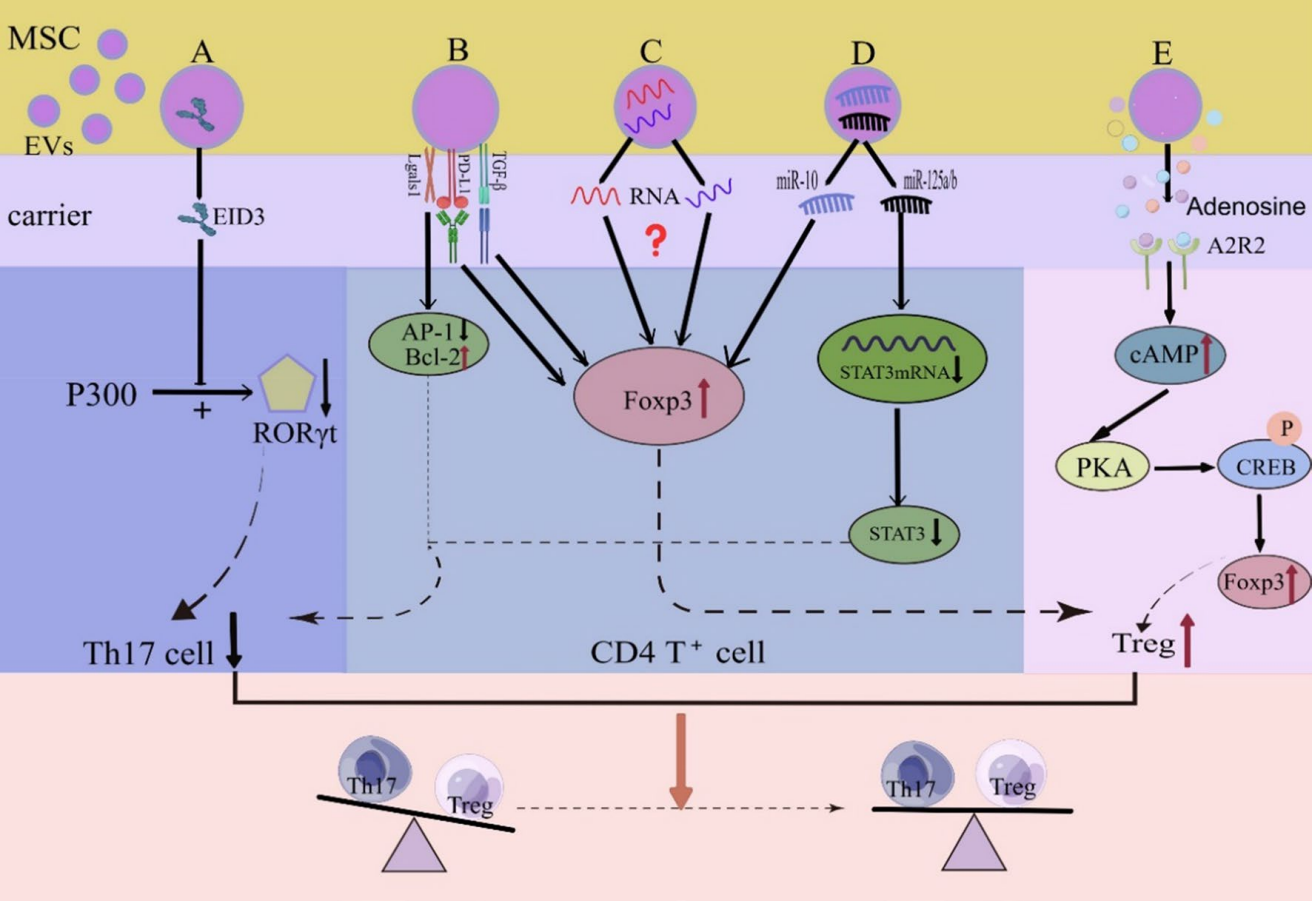
Schematic diagram of the main pathways and mechanisms by which extracellular vesicles (EVs) regulate Th17/Treg homeostasis**.** (By Figdraw.) **A** EID3: destabilizing the RORγt proteasome by inhibiting K63-linked ubiquitination and acetylase activity of p300, leading to degradation of the RORγt proteasome. **B** miRNA: miR-125a/b targets STAT3 mRNA and inhibits STAT3 expression, and miR-10 promotes FoxP3 expression. **C** Immune tolerance signaling molecules: PD-L1 and TGF-β induce FoxP3 expression, and Lgals1 activates the AP-1 transcription factor and downregulates Bcl-2 to induce effector T cell growth arrest and apoptosis.** D** RNA: An unknown RNA induces Treg production. **E** Immunometabolites: EVs are modified with adenosine packaging, and adenosine binds to A2AR on the Treg surface to activate intracellular cAMP levels, which in turn activates PKA and drives phosphorylation of cAMP response element binding protein (CREB), promoting Treg proliferation and immunosuppressive functions

Extracellular vesicles (EVs) are vesicles with a phospholipid bilayer secreted by almost all cell types [107]. The two main types of EVs, exosomes and microvesicles, are distinguished based on their biogenesis [108]. The biogenesis of exosomes occurs via the endocytosis-exocytosis pathway. First, the cell membrane invaginates to form early endosomes, which then interact with vesicles formed by the Golgi apparatus to form late endosomes. Late endosomes further develop into multivesicular bodies (MVBs) containing intracellular vesicles. The MVBs fuse with the lysosomal membrane or cell membrane and degrade, releasing the contents into the extracellular environment through exocytosis [109, 110]. However, microvesicles are formed by the external outgrowth of cell membranes in different cell types [110]. MSC-EVs are key immunomodulatory mediators of MSC signaling and can carry proteins, lipids, nucleic acids (DNA and miRNA), and soluble molecules [111]. MSC-EVs act on recipient cells by endocytosis, membrane fusion, and specific receptor-ligand recognition pathways, changing the phenotype, status, and function of recipient cells and inducing the differentiation of immune cells into more tolerant phenotypes or anti-inflammatory cells [112, 113]. Recent studies have reported that MSC-EVs maintain immune tolerance by modulating CD4^+^T cell subsets through multiple modalities (Fig. 2), attenuating the pro-inflammatory effects exerted by Th17 cells and enhancing the anti-inflammatory effects of Tregs as an effector mechanism [112, 114, 115]. Therefore, MSC-EVs are promising therapeutic agents.

A recent study showed that murine BM-MSC-EVs can inhibit Th17 cell differentiation by proteasomal degradation of RORγt via reduction of K63-linked polyubiquitination and acetylation, which contributed to the EP300-interacting inhibitor of differentiation 3 (Eid3) contained in the MSC-EVs [116]. This inhibition of Th17 cell differentiation is the mechanism by which MSC-EVs prevent Th17 cell differentiation from affecting post-translational modifications of RORγt proteins [116]. In addition, in a murine model for EAE, injection of MSC-EVs into mice inhibited IL-17 secretion and improved the clinical signs of EAE [116]. Yang et al. reported that IFN-γ-stimulated BM-MSC-EVs target Stat3 mRNA to inhibit Stat3 expression via miR-125a/b, thereby hindering the differentiation of Th17 cells in a colitis mouse model [117]. However, BM-MSC-EVs that were not stimulated by IFN-γ expression reduced the levels of miR-125a/b, suggesting that inflammatory factors can induce regulatory effects in MSC-EVs in the colitis mouse model [117]. Results showed that adipose tissue-derived MSC-EVs (ADSCs) promoted FoxP3 expression in naïve CD4^+^T cells and Treg cell generation, and interestingly, both RORγt and FoxP3 expression increased when miR-10 was loaded into ADSC-derived EVs [118]. This result seems to contradict the findings of the above study and may be related to the fact that the effects of MSC-EVs on various types of T helper cells vary depending on the experimental setting, including the origin of MSCs and environmental conditions. Moreover, Treg differentiation can be induced by modifying MSC-EVs, which are packaged with immunomodulatory metabolites such as adenosine, to bind to the adenosine receptor A2AR on the Treg surface under hypoxia-stimulated conditions [119]. Mokarizadeh et al. demonstrated for the first time that MSC-EVs can restore Th17/Treg homeostasis and reduce EAE model scores by carrying certain key molecules that mediate immune tolerance [120], such as PD-L1, galactose lectin-1 (Lgals1), and tolerance signaling molecules such as TGF-β. Specifically, PD-L1 expressed by MSC-EVs promoted Treg cell generation in EAE mice by inhibiting the Akt/mTOR signaling cascade, which enhanced and maintained FoxP3 expression [120]. Finally, human MSC-EVs promoted the conversion of EAE mice to a Treg anti-inflammatory phenotype. They reshaped immune homeostasis by inhibiting the secretion of Th17 cell-mediated pro-inflammatory cytokines or inducing the expression of Treg-related transcription factors and anti-inflammatory factors (e.g., FoxP3 and TGF-β) [121–123]. For instance, Koohsari found that infusion of EVs derived from human umbilical cord mesenchymal stem cells (hUCSC-EV) attenuated the severity of EAE mice by increasing the number of Tregs in the spleen of mice, reducing pro-inflammatory cytokines (IFN-γ, TNF-α, and IL-17A) in Th17 cells and upregulating anti-inflammatory cytokines (IL-10 and IL-4) [121]. Notably, deep RNA sequencing of IFN-γ-EVs revealed that IFN-EVs contain anti-inflammatory RNAs, and inactivation of some anti-inflammatory RNAs hindered the induction of Treg production in vitro [124]. This hindrance caused by the inactivation of some anti-inflammatory RNAs suggests that RNAs partially mediate the induction of Treg production, implying an important role of RNAs in the function of EVs [124].

Moreover, studies have shown that the inflammatory microenvironment is associated with the activity of biomolecules released by MSC-EVs, which mediate the regulatory effects of MSC-EVs on Th17/Treg homeostasis [117].

### Mitochondrial transfer

Mitochondria are crucial participants in cellular metabolism and energy homeostasis and are also important control switches that mediate the functional metabolism of CD4^+^T cell subsets [125, 126]. CD4^+^T cell activation and Th17 cell differentiation are mainly associated with increased glycolysis [127, 128], whereas Treg production is associated with mitochondrial lipid oxidization and pyruvate metabolism [129–133]. Interestingly, it was reported that a modality, mitochondrial kinetic effects, can mediate the immunomodulatory effects of MSCs on CD4^+^T cell subsets and demonstrated for the first time that Miro1 (a mitochondrial Rho-GTPase with a role in regulating mitochondrial movement from MSCs to recipient cells) modulates the transfer of MSCs to mitochondria via tunneling nanotubes (TNT) [134]. This modality altered the kinetics of CD4^+^T cells and modulated the phenotype and function of their subpopulations by targeting the mitochondrial network of CD4^+^T cells and their subpopulations [135, 136]. A recent study showed that adipose tissue-derived MSCs enhance the immunosuppressive function of Tregs by transferring active mitochondria and fragments of the plasma membrane to Tregs and that this transfer mode was dependent on MSC-expressed HLA and positively correlated with the HLA-C and HLA-DRB1 epitope mismatch load between Tregs and MSCs donors [137]. Angela et al. reported that MSC-mediated mitochondrial transfer induces Treg production by increasing the expression of FoxP3 miRNA, which was confirmed in a graft-versus-host disease (GVHD) model [138]. Furthermore, Jeong et al. demonstrated that CD39/CD73 signaling is an important factor driving the transfer of mitochondria from human marrow MSCs to Tregs, which promotes the immunosuppressive function of Tregs by increasing adenosine production in vitro [139]. Interestingly, UC-derived MSCs alleviate the energy starvation of CD4^+^T cells by transferring mitochondria to T cells by downregulating the autophagic process and apoptosis of CD4^+^T cells, which plays an important role in the treatment of systemic lupus erythematosus [140]. Luz-Crawford et al. reported that after co-culturing isolated expanded Th17 cells with human BM-MSCs for 4 h, the transfer of mitochondria from MSCs to Th17 cells resulted in a decrease in IL-17 secretion from Th17 cells and promoted the polarization of some Th17 cells into FoxP3 Treg cells to re-establish the Th17/Treg balance. This process alters the metabolic pattern of Th17 cells from glycolysis to oxidative phosphorylation, thereby suppressing the phenotype and function of Th17 cells and shifting it to the anti-inflammatory phenotype of Tregs [141].

Previous studies have shown that CD4^+^T cell mitochondrial disorders can disrupt their metabolic pattern in patients with MS, which can lead to disrupted differentiation of CD4^+^T cell subsets, thereby triggering a Th17/Treg skew towards Th17 cells and enhancing the inflammatory response in vivo [142–145]. This pathway provides an alternate perspective for exploring the mechanism of MSCs in MS therapy. It expands the therapeutic modality of stem cells and contributes to the transformation of MSC-based cell therapy into a novel therapeutic strategy targeting specific organelles.

### Metabolic reprogramming

Metabolic reprogramming is essential for the differentiation of CD4^+^T cell subsets and the regulation of Th17/Treg homeostasis [146–150]. Previous studies have shown that IFN-γ-stimulated mouse BM-MSCs could promote a metabolic switch in cellular metabolism from mitochondrial respiration to aerobic glycolysis. This aerobic state was dependent on the secretion of the immunosuppressive factors IDO and PGE2, suggesting that the energy metabolic pathway of MSCs mediates their immunomodulatory capacity [151, 152]. Elizabeth et al. reported that MSCs from human UC blood tissue that are driven by inflammatory cytokine inhibited mTOR signaling and HIF-1α gene expression in CD4^+^ T cells. This inhibition resulted in the inability of HIF-1α to bind to the promoter region of the RORγt gene and interfered with the glycolytic metabolic state of CD4^+^T cells, contributing to the polarization of CD4^+^T cells toward Treg and enhancing immunosuppression [153]. Contreras-Lopez et al. reported that the metabolism of peroxisome proliferator-activated receptor (PPARβ/δ) involved in fatty acid oxidation and glucose uptake pathways mediates the regulation of MSCs in the Th17/Treg homeostatic process in vitro [154]. The study found that MSCs lacking PPARβ/δ enhanced the inhibition of murine Th17 cell proliferation and induced Treg differentiation through enhanced glycolytic metabolism, accompanied by the production of immunomodulatory mediators (including IL-6, TGF-β1, and PD-L1) [154]. Likewise, in an in vitro study in which murine MSCs silenced with HIF-1α were co-cultured with murine naïve CD4^+^T cells, MSCs had a reduced potential to induce Th1 and Th17 cell production, which limited their ability to produce Tregs [155]. The authors further demonstrated that the reduced immunosuppressive potential of MSCs was associated with a metabolic switch from glycolysis to oxidative phosphorylation, and the production of several immunosuppressive mediators (including ICAM, IL-6, and nitric oxide) were associated with a reduced ability to produce some immunosuppressive mediators [155]. Furthermore, in a delayed-type hypersensitivity mouse model, murine MSCs expressing HIF-1α were again shown to reduce the frequency of pro-inflammatory Th17 cells and induce Treg cell production in vivo [155]. Notably, Yasufumi et al. reported that human BM-derived MSCs interact with human effector T cells via PD-1/PD-L1 to inhibit CD3z chain and Zap-70 phosphorylation, negatively regulate hexokinase II (HK2) protein expression, and suppress effector T cell glucose metabolism in vitro [156]. Although the phenotype of effector T cells was not further clarified, this suggests that PD-1/PD-L1 may mediate the immunomodulatory role of MSCs in the metabolic reprogramming of effector T cells. Therefore, from the perspective of metabolic reprogramming, further exploration should be conducted to determine whether PD-1/PD-L1 could act as a target for MSCs to regulate Th17/Treg homeostasis in the future.

In conclusion, for future MSC-based therapies, including EV and mitochondria, targeting cellular metabolism (including PPARβ/δ, mTOR/HIF-1α) has been and will be an attractive target for the development of alternate therapies.

### Autophagy

Autophagy is a fundamental mechanism for the protection of cellular homeostasis that is mediated by lysosomes and plays an integral role in maintaining bioenergetic homeostasis by controlling molecular degradation and organelle turnover [157–159]. Autophagy can be induced by starvation, inflammation, growth factor deficiency, and a variety of immune-related signaling molecules [157, 160]. Recent studies have shown that the regulation of MSC autophagy may be a novel mechanism that mediates the regulation of CD4^+^T cell subsets.

In an EAE mouse model, 3-methyladenine (3-MA) was shown to inhibit autophagy in MSCs, which activated the reactive oxygen species (ROS)-MAPK1/3 pathway in MSCs and subsequently induced the expression of prostaglandin-endoperoxide synthase 2 and downstream PGE2; this led to a reduction in the activation of CD4^+^T cells and attenuated the inflammatory response, ultimately improving the therapeutic effect of MSCs [161]. However, the numbers of Th17 cells and Tregs remained unchanged in another study, and therefore, results did not indicate that autophagy could regulate the differentiation of CD4^+^T cell subpopulations. Consequently, this study interpreted the improved treatment effect as a significant reduction in the activation and expansion of myelin-specific CD4^+^T cells [120].

Interestingly, the exact opposite finding was reported in another in vitro study, which showed that human BM-derived MSCs with activated autophagy (rapamycin pretreatment) enhanced MSC-mediated CD4^+^T cell differentiation through upregulation of TGF-β1 expression, thereby enhancing the immunosuppressive function of MSCs. In contrast, the use of 3-MA significantly attenuated the TGF-β1-dependent suppression of CD4^+^T cells by MSCs [162, 163]. Furthermore, compared with the control group, the experimental group showed an increased number of Tregs, a decreased proportion of Th1 cells, and reduced levels of pro-inflammatory cytokines, such as IL-17A, IFN-β, and IL-2 [163]. This outcome demonstrates that TGF-β1 plays a key role in the regulation of autophagy in MSCs, suggesting that TGF-β1 may be a target for mediating MSC therapy [163]. Thus, the induction of autophagy could be used to increase the production of TGF-β1 and several other immunosuppressive factors in MSCs, thereby significantly enhancing their therapeutic effects in immune cell-mediated diseases [163]. Notably, this approach has been demonstrated in the context of other autoimmune diseases, where infusion of rapamycin-induced adipose tissue-derived human MSCs into animals with acute GVHD (aGVHD) resulted in significantly reduced clinical manifestations of aGVHD compared with untreated animals. Moreover, the researchers found that the protective effect of autophagy activation was linked to increased production of immunosuppressive factors (TGF-β1, IL-10, and IDO) in MSCs in vivo and that MSC-derived IDO-induced enhanced Treg immunosuppression and was a key molecule in preventing Treg reprogramming into IL-17-producing effector Th17 cells [164]. In addition, the investigators found that mRNA expression of certain autophagy genes (such as autophagy-related 5 [ATG5] and light chain 3 [LC3]) was increased, suggesting that the activation of autophagy in adipose tissue-derived human MSCs before transplantation into animals with aGVHD suppresses Th17 cell production, induces Treg differentiation, and enhances Treg-mediated immune tolerance [164].

It is worth considering that several of the above experiments showed contradictory results, and the reasons behind these discrepancies are worth exploring. It can be explained in the following aspects: discrepancies can be attributed to differences in the species from which MSCs were obtained (mice and humans), cell culture conditions, and the inflammatory microenvironment surrounding the MSCs [165]. Alternatively, discrepancies may be related to autophagic flux [166], which is a measure of autophagic activity [166, 167]. Autophagy is a dynamic process that depends on the immediate cellular energy demand. In general, autophagy can be rapidly upregulated in response to environmental stresses, such as oxidative stress, starvation, hypoxia, inflammation, and infection, all of which have the potential to cause or exacerbate cellular damage [167, 168]. Activated autophagy constitutes a stress-adaptive pathway that promotes cell health and survival [167]. However, insufficient autophagy activation can reduce the degradation of defective organelles [165]. Conversely, overstimulation of autophagy can lead to cellular damage; more specifically, increased autophagy can lead to non-apoptotic forms of programmed cell death [169]. Stimulation of the inflammatory microenvironment is a prerequisite for MSCs to exert immunosuppressive effects [161, 170]. However, these conditions can also induce autophagy in MSCs and exhibit negative effects on their immunomodulatory activity [171]. In several of the above studies, researchers did not focus on measures of autophagic activity. This discrepancy may be partly attributed to the fact that autophagy acts as a negative feedback mechanism to balance the immune response [165]. Furthermore, autophagy may act as a double-edged sword, with its role changing depending on the characteristics, severity, and duration of the stressor [167]. In conclusion, the question of quantifying how the appropriate autophagic flux contributes to the regulation of Th17/Treg homeostasis by MSCs is a future research direction.

## MSCs for MS clinical research

MSC-based cell therapy has been applied clinically [41, 172–174] (e.g., Identifier: NCT00781872, NCT02034188, NCT01364246, NCT03326505, Table 2), and most clinical trials infused autologous BM-MSCs [173], with the first pilot study conducted in Iran in 2007 [175]. According to the literature, dozens of clinical trials have been registered for patients with MS and autologous or allogeneic MSCs from the BM, adipose tissue, and UC, with many reports involving early (phase I/II) clinical trials [176, 177] showing that intrathecal or intravenous MSC transplantation is feasible, safe, and tolerable, relieving clinical symptoms and reducing lesions. In particular, MSC infusion increases the levels of anti-inflammatory cytokines (IL-4 and IL-10) in the peripheral blood of patients with MS, a phenomenon that confirms the immunomodulatory effect of MSCs [177]. In a phase I clinical study conducted in Sweden on seven patients with MS, intravenous infusion of transplanted autologous BM-MSCs stabilized disability in 86% of patients during clinical remission [178]. Moreover, within one week after infusion, results showed an increase in the proportion of Tregs in the peripheral blood, suggesting an immune tolerance effect of MSCs in patients with MS [178]. Recently, Petrou et al. performed a phase II double-blinded trial in 28 men and 20 women with active progressive MS (Identifier: NCT02166021, Table 2) [173, 179]. This trial aimed to evaluate the optimal administration, safety, and clinical efficacy of autologous BM-MSC grafts in patients with active progressive MS. Additionally, compared to intravenous (IV) treatment and sham injections, the trial reported that patients with MS who received intrathecal MSC injections had significantly better scores on the timed 25-foot walk, 9-hole peg, and cognitive tests, as well as significantly improved relapse rates and lesion extent [173]. Furthermore, new results from a trial published in early 2022 showed that 60% of patients with MS treated with intrathecal autologous BM-MSCs had significantly lower CSF NF-L levels [180]. Interestingly, this effect was also observed in the group treated with IV MSCs, although this was not as pronounced as the intrathecal approach [180]. Thus, this trial suggests that MSCs are a viable therapeutic option for MS, with the best delivery method being intrathecal application. Moreover, an open-label phase I/IIa clinical study confirmed the feasibility and safety of autologous intrathecal BM-MSC administration in patients with SPMS and RRMS who failed to respond to conventional treatment (Identifier: NCT01895439, Table 2) [181]. Furthermore, compared to pre-treatment, a trend towards improvement was found in two patients with SPMS and intrathecal infusion of MSCs who showed a decrease of 4 and 3.5 points on the Expanded Disability Status Scale (EDSS), respectively [181].

**Table 2:** Summary of clinical trials with MSCs in treating multiple sclerosis

Clinical trial identifier	Clinical phase	Source	Registration year	Country	Clinical types in MS	Estimated number of MS patients	Primary evaluation after cell therapy	Ref./Completion year
NCT01730547	I/II	Autologous BM-MSCs	2012	Sweden	RRMS/SPMS/ PPMS	15	To assess the safety of IV therapy with autologous MSCs in MS patients	[178]
NCT01854957	I/II	Autologous MSCs	2013	Italy	Active MS	20	1.Safety (Incidence and severity of adverse events 2.efficacy (total number of contrast-enhancinglesions (GEL) at MRI scan)	[178]
NCT01606215	I/II	Autologous MSCs	2013	UK	Active MS	13	check the procedure is safe and to measure any changes on the MRI at 24 weeks	[178]
NCT02403947	I/II	Autologous BM-MSCs	2015	France	MS	12	Efficacy assessed by combined unique magnetic resonance imaging (MRI) activity, volume of GEL, and volume of BH (black holes)	[178]
NCT02035514	I/II	Autologous BM-MSCs	2013	Spain	RRMS	8	Safety and Cumulative number of MRI Gd-enhancing lesions	[178]
NCT00813969	I	Autologous MSCs	2011	USA	RMS	24	To evaluate the feasibility of culturing MSCs, and infusion-related safety and tolerability of autologous MSC transplantation over one month in patients with relapsing forms of MS	[179]
NCT02166021	II	Autologous BM-MSCs	2014	Israel	Active progressive MS	36	To evaluate brain MRI and Immunological Response	[169]
NCT00395200	I/II	Autologous BM-MSCs	2006	UK	MS	20	Establish the safety by monitoring adverse reactions	(180)
NCT00781872	I/II	Autologous BM-MSCs	2006	Not Provided	MS	20	Safety and migration ability of the injected cells, clinical efficacy	[181]
NCT00014755	I	Autologous BM-MSCs	2001	USA	MS	35	To evaluate brain MRI, CSF, Long-term complications and survival	[182]
NCT01056471	I/II	Autologous AD-MSCs	2011	Spain	SPMS	30	Infusion of autologous AD-MSCs is safe and feasible in patients with SPMS	[183]
NCT03326505	I/II	Allogenic UC-MSCs	2017	Jordan	MS	60	The number, intensity and volume of CNS lesions will be assessed to investigate the therapeutic benefits of the injected Allogenic MSCs and/or Physical therapy by MRI	[184]
NCT02034188	I/II	Allogenic UC-MSCs	2014	Panama	MS	20	No serious adverse events were reported. MRI scans of the brain and the cervical spinal cord showed inactive lesions in 15/18 (83.3%) subjects after 1 year	[185]
NCT01377870	I/II	Autologous BM-MSCs	2011	Iran	RRMS	30	Evaluate the effect of MSC transplantation on number of Gd(gadolinium) positive lesions	2014
NCT01895439	I/IIA	Autologous BM-MSCs	2013	Jordan	MS	30	By Magnetic Resonance Imaging (MRI) and ophthalmological tests	2017
NCT02239393	II	Autologous MSCs	2014	Canada	RRMS/SPMS/PPMS	40	1.Safety (Incidence and severity of adverse events 2.efficacy (total number of contrast-enhancinglesions (GEL) at MRI scan)	2014
NCT01056471	I/II	Autologous AD- MSCs	2010	Spain	SPMS	30	To evaluate safety and tolerability related to the intravenous infusion of autologous mesenchymal stem cells	2015
NCT00017628	I	Autologous MSCs	2001	USA	MS	20	MSC therapy is safe without side effects after injection	2005
NCT03069170	I	Autologous BM-MSCs	2017	Jordan	RRMS	50	Effectiveness assessment by MRI Safety assessment by physical examination,vitalsigns,analytical results,electrocardiograph monitoring, and EDSS	Unknown
NCT02495766	I/II	Autologous BM-MSCs	2015	Spain	RRMS/SPMS	8	MS therapy is safe without side effects after cell injection. Evaluated EDSS score	2018
NCT02157064	Not specified	Autologous AD-MSCs	2014	USA	MS	100	Change from Baseline on the Multiple Sclerosis Quality of Life Inventory (MSQLI) at 12 months	Unknown
NCT01364246	I/II	Allogenic UC-MSCs	2010	China	Progressive MS	20	Evaluated core of EDSS, VEP (visual evoked potential), MRI, SEP (somatosensory evoked potential) and BAEP (brainstem auditory evoked potential). No side effects were apparent after cell injection	2014

*AD* adipose tissue, *BM* bone marrow, *UC* umbilical cord, *MSCs* mesenchymal stem cells, *MS* multiple sclerosis, *RRMS* relapsing–remitting multiple sclerosis, *PPMS* primary progressive multiple sclerosis, *SPMS* secondary progressive multiple sclerosis, *EDSS* expanded disability status scale

In addition to the above clinical studies, other clinical trials conducted to date are summarized in Table 2 [182–189]. We noted that, first, current clinical trials mostly focused on phase I/II studies. The sources of MSCs included BM, adipose tissue (AD), and UC. Most of the studies focused on safety and efficacy after transplantation. Second, the outcome metrics are mostly focused on EDSS score and magnetic resonance imaging. From the available studies, most of the trials showed favorable safety outcomes and a few minor side effects, including fever, headache, urinary tract infection, and respiratory tract infection. Additionally, it was found that multiple infusions of MSCs produced beneficial effects and that infusion time is another important factor. Previous studies have also shown that the therapeutic effect of MSCs is closely related to the stage of EAE disease [190]. Murine BM-MSC infusion significantly reduced the percentage of Th17 cells. It upregulated the percentage of Treg cells during the early stages of EAE progression, but the immunosuppressive capacity of MSCs during the stable phase was not significantly changed [190, 191]. This lack of significant change may be related to the plasticity of MSCs, as the inflammatory microenvironment is crucial for their immunosuppressive functions [81]. Thus, an accurate assessment of patients’ inflammatory status and selection of an appropriate time point for MSC infusion is crucial for the treatment of MS [191]. Although no direct clinical trials are focusing on whether MSCs inhibit Th17 cell production, current clinical studies have shown that MSCs can induce an increase in the Treg ratio and restore the immune tolerance status in patients with MS [178]. In addition, pre-clinical studies have indicated that MSCs limit Th17 cell proliferation and promote Treg production and immunosuppressive capacity, suggesting that MSCs have the potential to re-establish the Th17/Treg balance in clinical applications of MS [81] (Table 3).

**Table 3:** The effect of current immunomodulatory drugs on Th17/Treg homeostasis

The type drugs	Effects of drugs on Th17/Treg balance on MS/EAE	Mechanisms of drugs on Th17/Treg balance	References
IFN-β	Inhibit the secretion of pro-inflammatory IL-17 in MS	Suppress IL-17 secretion by T cells via IFN-α/β receptor signaling	[22]
GA	Target the Th17 cell population by inhibiting the production of IL-17 and promote Treg production inMS	Activate Foxp3 which promotes the development of CD4 + CD25 + Tregs	[23]
S1PR	Decrease secretion of pro-inflammatory IL-17 by Th17 cells in MS	Delete the S1P1 in Th17 cells	[29]
Laquinimod	Impede Th17 proinflammatory response and promoting secretion of anti-inflammatory IL-4 and IL-10 cytokines	Downregulate the VLA-4 mediated lymphocytes adhesiveness	[26]
DMF	Shift inflammatory responses from Th17/ Th17 to Th2, resulting in decreased IL-17 and IFN-γ producing CD4 cells Reduce relative and absolute numbers of Th17 cells	Down-regulate the pattern of glycolytic metabolism that contributes to Th17 cell generation	[25]
Teriflunomide	Reduce the absolute numbers of Th1, Th17 and Th17.1 cells	Inhibit the dihydro-orotate dehydrogenase enzyme required for de novo pyrimidine synthesis in lymphocytes	[27]
Rituximab	Decline of Th1 and Th17 in the periphery and within the CNS of EAE	Hamper Th17 cells by direct (depletion) and indirect (reduced activation by B cells)	[28]
Cladribine	Downregulation the Th17 cell population	Disrupt DNA synthesis by inhibiting enzymes involved in the cell cycle	[24]

*GA* glatiramer acetate, *IFN-β* interferon-beta, *DMF* dimethyl fumarate, *SIPR* sphingosine 1 phosphate receptor

## Use of engineered and preconditioned MSCs in MS experimental models

MSCs are highly plastic, and pretreatment and engineering modification of MSCs with biological, chemical, or physical factors has been shown to be an effective strategy for enhancing their therapeutic functions in EAE mice [192, 193].

There are numerous ways to pretreat MSCs. For example, UC-MSCs pretreated with IFN-γ enhanced their secretion of indoleamine 2,3- dioxygenase1 (IDO1), decreased serum IL-17A and TNF-α levels, and ultimately improved clinical signs in EAE mice [193]. In addition, pretreatment with CXC cytokine member stromal cell-derived factor 1α (SDF-1α) increased C-X-C chemokine receptor type 4 (CXCR4) expression on the surface of BM-MSCs and improved myelin regeneration in the brassinosteroid model. Tetramethylpyrazine (TMP) pretreated UCMSCs improved the clinical severity of EAE and reduced clinical scores, inflammatory cell infiltration, NLRP3 levels, demyelination, and BBB disruption [194]. Results have shown that EAE rats treated with MSCs pretreated with 17β-ED decreased the gene expression of pro-inflammatory cytokines IL-17, TNF-α, and IFN-γ, as well as MMP8 and MMP9. In contrast, it elevated the anti-inflammatory cytokines IL-10, IL-4, and TGF-β [195]. Altogether, these results suggest that pre-treatment may be an important factor in enhancing the immunosuppressive properties of MSCs, which may improve cell survival and immunomodulatory functions. Similarly, engineered modifications of MSCs have increased the therapeutic potential of MSCs. A study showed that transduction of IFN-β into AD-MSCs decreased IL-17 expression and induced Tregs and IL-10 production in EAE mice, which ultimately reduced the clinical score and inflammatory cell infiltration [196]. In addition, transfection modification of MSC with triple P-selectin glycoprotein ligand-1 (PSGL1)/sialic acid-Lewis/IL-10 mRNA reduced clinical scores and inflammatory infiltration of the spinal cord in EAE mice [197]. Additionally, a report showed that UC-MSCs transfected with the sphingosine kinase 1 (SPK1) gene reduced pro-inflammatory cytokines and increased Treg cell production in the serum of EAE mice. This transfection also led to a reduction in the infiltration of inflammatory cells and the degree of demyelination [198].

Most of these current in vitro treatments are based on pre-clinical studies and have shown promising results. However, whether these strategies can be translated into clinical studies needs to be further explored to improve the therapeutic efficacy of transplanted MSCs in the clinically relevant setting of MS and other immune-mediated CNS diseases.

## Conclusion

MSCs regulate Th17/Treg homeostasis through extracellular vesicles, metabolic reprogramming, mitochondrial transfer, autophagy, and other pathways to restore immune self-stabilization and the tolerance state, ultimately attenuating the degree of neuroinflammation and demyelination in MS/EAE in vivo. Given the tight connection between cellular metabolism and immunoregulatory networks, molecules involved in mitochondrial translocation and metabolic reprogramming pathways (including Miro1 and PPARβ/δ) may be potential targets for MSCs to regulate immune homeostasis. Furthermore, the increasingly popular EV and autophagic pathways have emerged as new mechanisms for MSCs to regulate the Th17/Treg balance. EVs not only efficiently cross the BBB but also contain a variety of contents (including miRNAs, proteins, etc.) with immunomodulatory effects. However, studies on the contents of EVs remain relatively scarce. In addition, the immunomodulatory capacity of MSCs seems to correlate with the level of autophagy activation, but precise modulation of the degree of autophagy to determine the optimal regulatory equilibrium deserves further exploration (e.g., a measure of autophagic flux: LC3, etc.). There remain some knowledge gaps in the mechanisms by which MSCs regulate the Th17 / Treg balance, and further research is needed to translate the mechanisms into clinical therapy. Finally, future clinical studies should focus on the optimization of pre-treatment and engineered modifications, infusion time points, infusion doses, and methods of administration to enhance the effectiveness of MSCs in treating MS and other autoimmune CNS diseases.

## Data Availability

Date are available by emailing the corresponding author.

## Abbreviation

MSCs: Mesenchymal stem cells
MS: Multiple sclerosis
RRMS: Relapsing–remitting multiple sclerosis
PPMS: Primary progressive multiple sclerosis
SPMS: Secondary progressive multiple sclerosis
BBB: Blood–brain barrier
DMT: Disease-modifying therapies
GA: Glatiramer acetate
EAE: Autoimmune encephalomyelitis
EVs: Extracellular vesicles
CNS: Central nervous system
BM: Bone marrow
UC: Umbilical cord
AD: Adipose tissue
IDO1: Indoleamine 2,3- dioxygenase1
CSF: Cerebrospinal fluid
DMF: Dimethyl fumarate
sTNFR1: Soluble TNF receptor 1
MMP: Matrix metalloproteinase
MCP-1: Monocyte chemotactic protein 1
PD-L1: Programmed death ligand-1
Lgals1: Galactose lectin-1
TNT: Tunneling nanotubes
GVHD: Graft-versus-host disease
aGVHD: Acute graft-versus-host disease
HK2: Hexokinase II
SLE: Systemic lupus erythematosus
DTH: Delayed-type hypersensitivity
3-MA: 3-Methyladenine
EDSS: Expanded disability status scale
GA: Glatiramer acetate
IFN-β: Interferon-beta
DMF: Dimethyl fumarate
SIPR: Sphingosine 1 phosphate receptor
